# Selection of Aptamers Specific for Adipose Tissue

**DOI:** 10.1371/journal.pone.0037789

**Published:** 2012-05-25

**Authors:** Jun Liu, Huixia Liu, Kwame Sefah, Bo Liu, Ying Pu, Dimitri Van Simaeys, Weihong Tan

**Affiliations:** 1 Division of Geriatrics, Xiangya Hospital, Central South University, Changsha, Hunan, China; 2 Center for Research at Bio/Nano Interface, Department of Chemistry and Department of Physiology and Functional Genomics, Shands Cancer Center, UF Genetics Institute and McKnight Brain Institute, University of Florida, Gainesville, Florida, United States of America; Consejo Superior de Investigaciones Cientificas, Spain

## Abstract

**Background:**

Obesity has reached epidemic proportions, affecting more than one tenth of the world’s population. As such, adipose tissue is being increasingly recognized as an important therapeutic target for obesity and related metabolic disorders. While many potential targets of adipose tissue have been established and drugs developed, very few of those drugs specifically target adipose tissue without affecting other tissue. This results from a limited knowledge of both cell-surface markers and physicochemical traits specific to adipocytes that might otherwise be exploited by circulating drugs.

**Methodology/Principal Findings:**

Here we report the use of cell-SELEX technology to select two aptamers that can specifically recognize mature adipocytes: adipo-1 and adipo-8. Adipo-8 shows high affinity for differentiated, mature 3T3-L1 adipocytes with a K_d_ value of 17.8±5.1 nM. The binding was sustained upon incubation at 37°C and insulin stimulation, but was lost upon trypsin treatment. The binding ability was also verified on frozen tissue slides with low background fluorescence and isolated adipocytes.

**Conclusions/Significance:**

Aptamer adipo-8 selected from a random library appears to bind to mature differentiated adipocytes specifically. This aptamer holds great promise as a molecular recognition tool for adipocyte biomarker discovery or for targeted delivery of molecules to adipocytes.

## Introduction

Obesity has reached epidemic proportions in most industrialized countries, leading to an increased prevalence of metabolic syndrome characterized by visceral obesity, hypertension, dyslipidemia, and insulin resistance. Adipose tissue has long being viewed solely as a site for pure fat storage. However, during the last 20 years, adipokines, or adipocytokines, which are cell-to-cell signaling proteins secreted by adipose tissue, have been studied and characterized, leading to a surge of scientific interest in adipocytes. Adipose tissue is primarily composed of adipocytes, which are cells that store fat as energy, but adipocytes also have an endocrine function in regulating energy homeostasis and serving as an integrator of various physiological pathways [1], [2], [3].

Many studies have demonstrated that obesity, particularly visceral obesity, is mainly involved in increasing the clinical risk of metabolic and cardiovascular diseases [4], [5]. Visceral adipose tissue is now thought to play a pivotal role in metabolic syndrome and its clinical consequences [6], [7]. Targeting adipose tissue as a way to treat these diseases has been investigated and tested [8]. For example, peptide motif homing to white fat vasculature has been developed using the phage display method. Conjugation with pro-apoptotic peptide [9] and 11β-hydroxysteroid dehydrogenase type 1 (11β-HSD1) inhibitor [10] has also shown some promising results.

Targeted delivery of drugs specific to adipose tissue has also been envisioned as an effective method to treat metabolic disorders [8], [11], [12]. However, while many potential targets of adipose tissue have been established and candidate anti-obesity drugs have been developed, none of those drugs specifically targets adipose tissue, leading to undesirable side effects and lower efficacies. This results from a limited knowledge of both cell-surface markers and physicochemical traits specific to adipocytes that may otherwise be exploited by circulating drugs.

Cell-SELEX is an established method used to select short strands of nucelotides, called aptamers, which are able to recognize target cells with high binding affinity and specificity. Currently, aptamers have been generated from several cell lines using this technique, including leukemia cells [13], glioblastoma cells [14] and lung cancer cells [15]. Aptamers generated from this method also provide an alternative tool for the discovery of cell-surface biomarkers that can distinguish target cells from other cell lines [16], [17], [18]. Moreover, aptamers can be easily engineered to carry drug payloads, e.g., siRNAs (14). Given the ability of siRNAs to knock down any gene of interest [19], turning off gene activity via siRNAs could have therapeutic benefits. Drugs delivered in this way will significantly enhance efficacy of drug therapy, while avoiding side effects by unwanted action on other cell types.

By using 3T3-L1, as a positive adipocyte cell line, and both its precursor preadipocyte and HepG2 as negative cell lines, we have selected an adipocyte-specific aptamer using cell-SELEX technology. This aptamer, denoted adipo-8, binds with high affinity only to differentiated adipocytes, but not to preadipocytes or other cell lines tested. By its ability to specifically recognize mature adipocytes, this aptamer may also serve as a tool for adipocyte biomarker discovery or even targeted delivery of drugs for the treatment of adipocyte-related metabolic disease.

## Results and Discussion

### Enrichment of Aptamer Candidate Using Cell-SELEX

As described in Methods, cell-SELEX was used to select aptamer candidates from a random library against the mature adipocyte cell line 3T3-L1, a classical cell line for the study of adipocytes *in vitro*. Upon differentiation, preadipocytes transform from fibroblast-like cells into mature lipid-containing adipocytes that share many characteristics of adipose cells *in vivo*. To exclude aptamer candidates which bind to widely expressed membrane proteins and their precursor preadipocytes, liver cancer cell line HepG2 and undifferentiated preadipocytes were used for counter selection.

The selection process was initiated by incubating 20 pmol of library ssDNA’s with mature 3T3-L1 cells on a culture plate. After washing, cells were collected, and those sequences binding to target were eluted by heating the collecting solution to 95°C. Sequences that bound to general cell surface targets were removed by incubating the enriched pool with undifferentiated 3T3-L1 cells (rounds 2,4,6,8,10 and 12–19) and HepG2 cells (rounds 3,5,7,9 and12). After each round, the eluted pool was PCR-amplified, and the ssDNA recovered from the PCR product was used for the next round of selection or for flow cytometry to monitor pool enrichment. In contrast to other cell lines, differentiated 3T3-L1 adipocytes appeared as scattered dots in Side Scatter (SSC) and Forward Scatter (FSC) flow cytometry, resulting from the speed of adipogenesis among individual cells. In the dot plot, SSC reflects the granularity of the cell, while FSC reflects the cell size. As reported in the literature, 3T3-L1 cells will move upward in SSC after differentiation into mature adipocytes duo to increased cytoplasmic granularity caused by lipid droplet accumulation [20], [21]. Therefore, we set a cutoff point at ∼200 so that a dot plot above this value would be regarded as reporting differentiated, mature adipocytes (Figure S1), while below this value, only undifferentiated cells or cell debris were expected.

**Figure 1:**
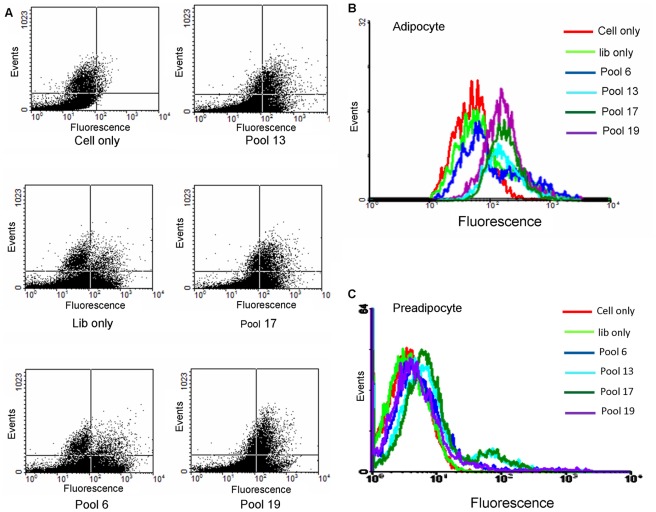
Flow cytometry results for selected pools (500 nM) with differentiated 3T3-L1 adipocyte and preadipocyte cells. (A) Dot plot of flow cytometry with selected pools. (B) Flow cytometry assay to monitor the pool enrichment for differentiated 3T3 adipocytes. (C) The slight shift of the respective pools to control 3T3-L1 preadipocyte was recovered by counter selection.

After a repeated positive-negative selection process, we observed a clear dot plot shift from the original pool in flow cytometry by incubating the pool enriched from the 13^th^ round with detached adipocytes. In the dot plot Figure 1A, only a few cells appeared in the upper right quadrant. When library pool was added to the cells, we noticed a clear cell population in the left upper quadrant. However, from the 6th to the 13th round pool, the cell population shifted to the upper right quadrant. This tendency was clearly illustrated in the histogram after gating; there was a clear shift from left to right and from the 6th to the 16th pool. On the other hand, from the 16^th^ to the 19^th^ pool, no further shift was noticed (Figure 1B). These results indicated that the pool was successfully enriched for sequences which bind to membrane targets expressed by the mature adipocyte. These pools were then testing for binding to the negative cell lines. None of the enriched pools could bind to preadipocytes (Figure 1C) or HepG2 cells (Figure S2). Pool signals were also reduced relative to background in the preadipocytes. Since most pool sequences appeared to bind only surface markers expressed in mature 3T3-L1 adipocytes, the pools generated from rounds 13, 17, and 19 were gel-purified and sequenced.

### Sequences Selected for Synthesis and Tests for Binding Ability

**Figure 2:**
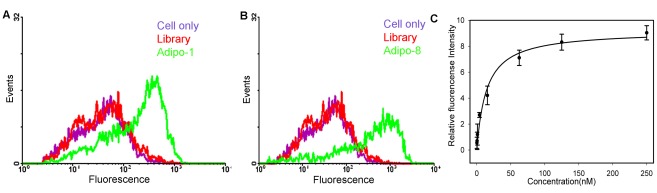
Characterization of selected aptamers. Flow cytometry for the binding of PE/cy5-labled adipo-1 (A) and adipo-8 (B) to differentiated 3T3-L1 adipocytes at a concentration of 250 nM. (C) K_d_ determination for adipo8. Adipocytes were incubated with varying concentrations of PE-Cy5-labeled adipo8 aptamer. The relative fluorescence intensity represents a ratio obtained through the following formula: relative fluorescensce intensity =  [(fluorescensce of aptamer - fluorescensce of library)/fluorescensce of library]. Error bars represent standard deviations. Each data point was performed in triplicate.

Analysis of the enriched pools from rounds 13, 17, and 19, indicated that they could all be categorized into 8 families based on their sequence homology. Eight sequences showing highest homology among the different pools were regarded as aptamer candidates and synthesized. To further test the binding ability of individual synthesized aptamers, biotin was coupled to the 5′ end of each aptamer candidate and streptavidin-PE was added for flow cytometry. Among eight families tested, aptamers adipo-1 and adipo-8 had the strongest binding (Figure 2A, B and Figure S3). Detailed sequences are shown in Table 1. Aptamers (250 nM) were also tested with control cells, and flow cytometry showed no binding for either aptamer to preadipocytes or HepG2 cells.

**Table 1:** Detailed sequences of aptamers used in the binding assay (primers are blue-colored).

Name	Sequence
**Adipo-1**	**ATGAGAAGCGTCGGTGTGGTTACTCCGGGCACTTGATATATCGATATGGAGAATAATGCACCCTGAGCGGGCTGGCAAGGCGCATA**
**Adipo-8**	**ATGAGAAGCGTCGGTGTGGTTAAACACGGAACGAAGGTGCAGGAAGATTTGTCGATGCGGTGCCTGAGCGGGCTGGCAAGGCGCATA**

**Figure 3:**
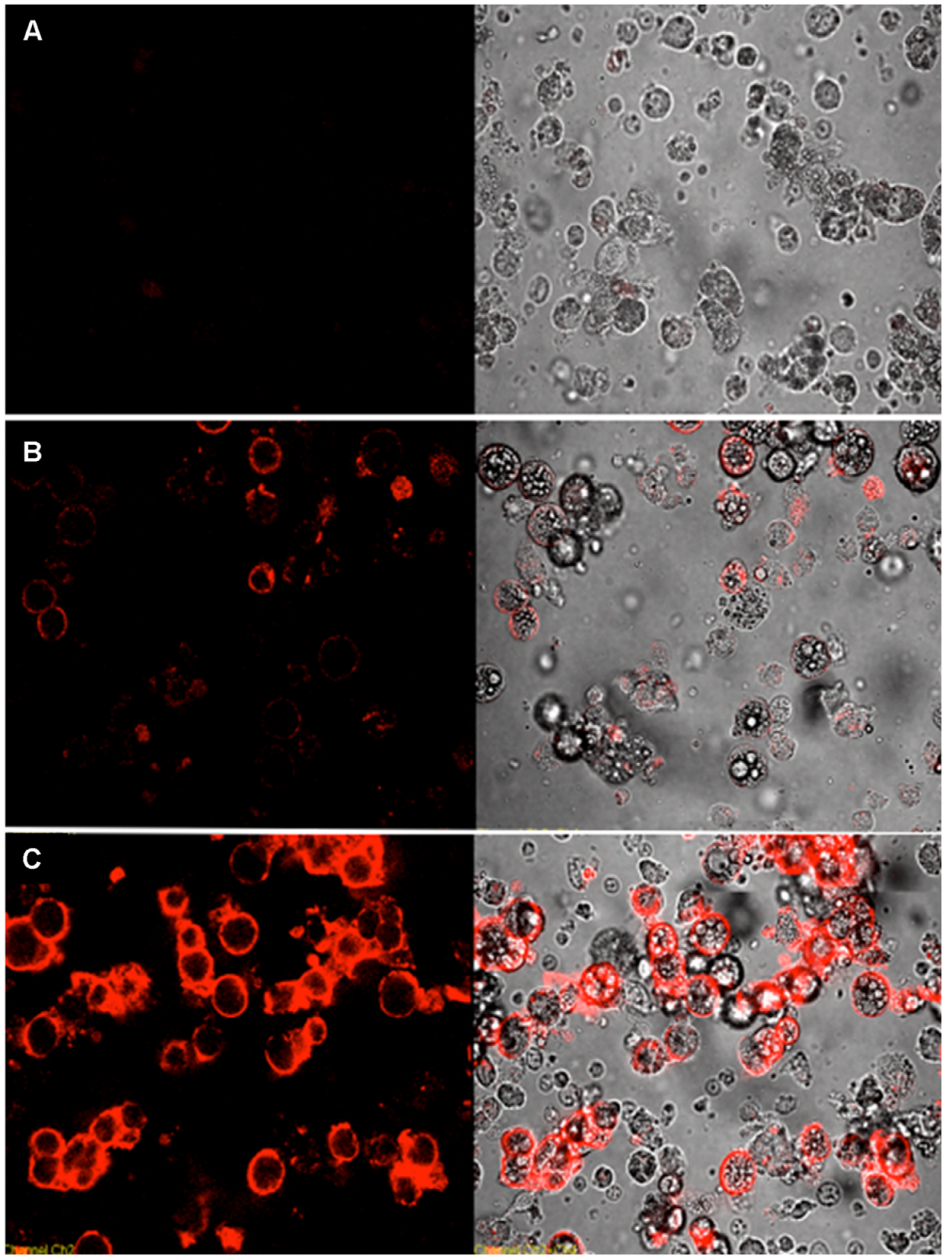
Confocal images of aptamers stained with cultured differentiated 3T3-L1 adipocytes. Cells were incubated with aptamers conjugated with biotin, and binding events were observed with PE-conjugated streptavidin. (A) unselected PE-labeled library; (B) adipo-1; (C) and adipo-8. The final concentration of the aptamers in the binding buffer was 250 nM. *(Left*) fluorescence and (*Right*) optical images of differentiated 3T3-L1 adipocytes.

As shown in Figure 2C, the binding K_d_ was also determined. The calculated K_d_s of adipo-1 and adipo-8 to mature adipocytes were 97.5±13.5 nM and 17.8±5.1 nM, respectively. The binding of these two aptamers to adipocytes was also verified by confocal microscopy. As shown in Figure 3, almost no signal was observed when initial library was incubated with the differentiated adipocytes. However, when adipo-1 was incubated with cells, we observed a round red signal located in the peripheral area of the lipid-containing adipocytes. The signal became much stronger when adipo-8 was incubated with mature adipocytes, indicating that adipo-8 binds more strongly than adipo-1 to mature adipocytes.

### Specificity of Selected Aptamers

Next, the specific binding of the aptamers selected against 3T3-l1 adipocytes was tested against the following cell lines: A549 (human lung adenocarcinoma epithelial cell line), Hct 116 cells (colorectal carcinoma HCT-116 cells), human B cells, T cells, CK562 (human erythroid leukemia), Ramos (human Burkitt’s lymphoma cell line), PL45 (pancreatic cancer cells), MCF-7(breast cancer cells) and A172 (human glioblastoma cell line). As shown in Table 2, none of these cell lines showed binding to adipo-8, while a slight shift could be observed in the A549, PL45, and MCF-7 cell lines with adipo-1. After 3T3-L1 preadipocytes differentiate into adipocytes, the following known proteins are expressed on the cell membrane: GLUT4 [22], fatty acid transport protein (FATP1) [23], fatty acid translocase (FAT/CD36) [24], plasma membrane fatty acid binding protein (FABPpm) [25], Insulin-responsive aminopeptidase (IRAP) [26], transferrin receptor (TfR) [27], and Caveola and its proteins [28]. Since all of these proteins are also expressed on either hepatocytes or muscle cells, we next tested the binding ability of adipo-8 to hepG2 cells, WT hepatocytes, and differentiated C2C12 cells (muscle cells). Again, however, no binding shift could be observed, demonstrating that the selected aptamer adipo-8 had high specificity to mature 3T3-L1 adipocytes.

**Table 2:** Relative binding of aptamer adipo-1 and adipo-8 with different cell lines.

	3T3-L1	A549	Human B cell	Human T cell	Hct 116	CK562	Ramous	PL45	MCF-7	A172	HepG2	WT hepatocytes	C2C12
**Adipo-1**	++	+	−	−	−	−	−	+	+	−	−	−	−
**Adipo-8**	+++	−	−	−	−	−	−	−	−	−	−	−	−

### Characterization of Selected Aptamers

**Figure 4:**
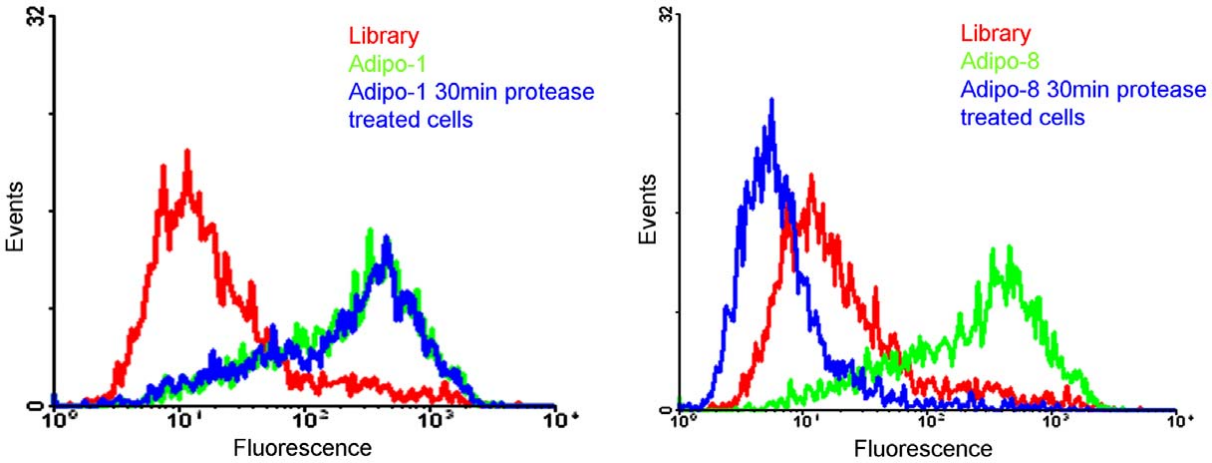
Preliminary determination of the nature of the molecular targets to which aptamers bind. Effect of trypsin treatment on the binding ability of adipo-1 (A) and adipo-8 (B) to differentiated 3T3-L1 adipocytes. The concentration of aptamers in the binding buffer was 250 nM.

**Figure 5:**
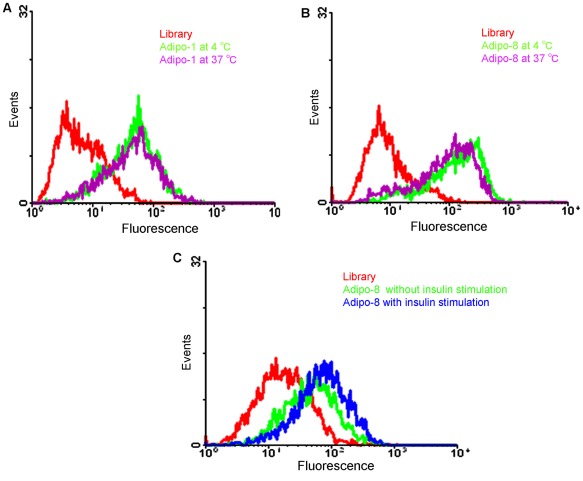
Flow cytometry assays to assess if aptamers’ binding ability to mature adipocytes can be influenced by temperature and insulin stimulation. Effects of temperature (A, B) and insulin stimulation (C) on the binding ability of adipo-1 and adipo-8 (250 nM) to differentiated 3T3-L1 adipocytes.

Two broad-spectrum serine proteases, trypsin and proteinase K, with different mechanisms of action, were also used in the binding studies of adipo-1 and adipo-8. After treatment of mature adipocyte with trypsin for 30 min, there was no significant shift back observed with adipo-1. However, the same treatment with adipo-8 did result in a significant flow cytometry shift back (Figure 4), indicating that the target molecule binding with adipo-8 is most likely some kind of protein. In addition, since selection processes are generally performed at 4°C, we also tested whether physiological temperature would affect the binding ability. Aptamers adipo-1 and adipo-8 were incubated with target cells at 37°C and 4°C, but no differences in binding ability were observed at either temperature (Figure 5A,B).

Most currently known proteins expressed on the adipocyte membrane surface after differentiation are responsive to insulin stimulation. For example, proteins like GLUT4, FATP1, IRAP have ten-fold increased expression in the cell membrane upon insulin stimulation, while other proteins like FAT/CD36, FABPpm, TfR increase two- to three-fold [29], [30], [31], [32]. Consequently, we tested whether insulin stimulation would affect the binding ability of adipo-8. Serum starvation was performed before the experiment to increase cellular response to insulin [33]. Cells were then treated with insulin at a concentration of 160 nM before flow cytometry. However, no significant shift changes were observed with or without insulin stimulation (Figure 5C), indicating that this aptamer may bind to a protein that is not involved in the insulin signaling pathway and, further, that it may be a possible adipocyte biomarker not yet reported.

### Binding of Selected Aptamer Adipo-8 to Frozen Adipose Tissue

**Figure 6:**
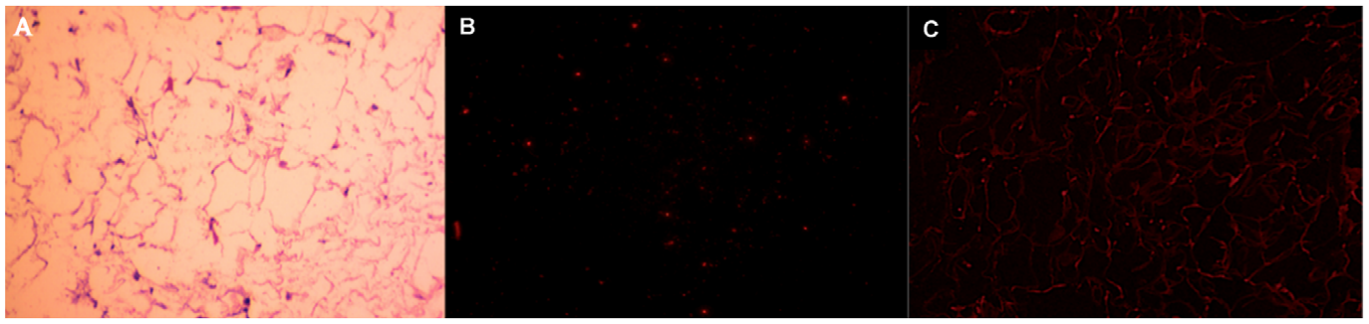
(A) HE staining of frozen adipose tissue from SD rats. (B) Adipose tissue frozen sections stained with 200 nM Cy5-labeled library (250 nM) (C) Adipose tissue frozen sections stained with 200 nM Cy5-labeled adipo-8 (250 nM).

Since the selected aptamers specifically bound to mature adipocytes, adipo-8 was tested to determine if it recognized adipose tissue in a real clinical sample. To maximally preserve the antigens at their original state on the adipocyte cell membrane, epididymal adipose tissue was removed immediately after sacrifice of the rat and used for frozen slide. After fixing with cold acetone for 10 min, the frozen slide was used for staining with cy5-labled adipo-8 and cy5-labeled library control. The results of fluorescence imaging and H&E staining are illustrated in Figure 6. Although a very strong fluorescence was noted with adipose tissue, very limited background fluorescence could be observed when cy5-labeled library was incubated with the adipose tissue slide, a result which is contrary to a previous report of high background fluorescence when aptamers were incubated with formalin-fixed, antigen-retrieved tissue [34], [35]. We have also extended the binding test to frozen slides from liver, skeletal muscle, pancreatic tissue, and no fluorescence was observed (Figure S4). To explain this inconsistency, it is proposed that the extensive intra- and intermolecular crosslinks of tissue proteins induced by formalin may increase the chance of nonspecific binding. Further investigation is needed to evaluate this phenomenon by comparing the effect of various modes of treating tissue sections for binding ability.

### Binding of Selected Aptamer Adipo-8 to Isolated Rat Adipose Cells

**Figure 7:**
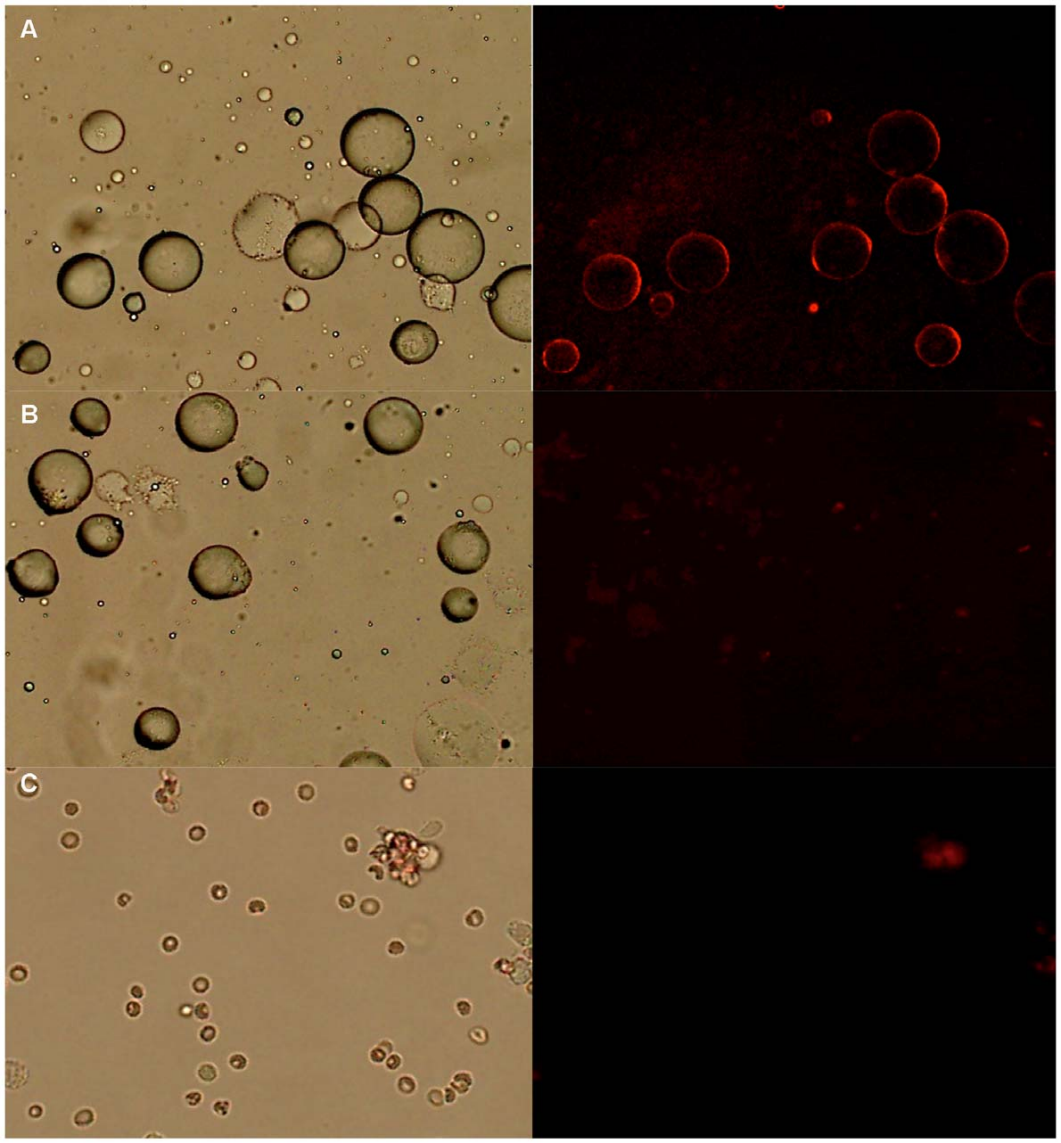
Molecular recognition of adipo-8 with isolated adipocyte. Fluorescence image of isolated mature adipocyte stained by cy5-labled adipo-8 (A), library (B). Fluorescence image of isolated pelleted cells of from collagenase-digested adipose tissue stained by cy5-labled adipo-8 (C). The final concentration of the aptamers in the binding buffer was 250 nM. Optical (Left) and fluorescence (Right) image of adipocyte and pelleted cells.

Since the aptamer adipo-8 selected from 3T3-L1 cell line shows binding to adipose tissue from S-D rats, we then investigated whether this aptamer can bind with isolated adipocytes. The adipose tissue contains different cells including adipocytes, preadipocytes, fibroblasts, as well as tissue resident macrophages, and vascular constituents [36]. Primary adipocytes were then isolated from epididymal fat pads using methods described by Sugihara [37]. The adipocytes can be easily separated from other cell lines due to their floating properties after centrifugation. We then tested the binding of cy5 labeled adipo-8 and the unselected ssDNA library with floating adipocytes and pelleted cells from digested adipose tissue separately. After incubation with the floating adipocytes for 45 min, we observed strong fluorescence under fluorescence microscopy, compared to the weak background fluorescence observed after incubation with the control cy5-labled ssDNA library (Figure 7). Furthermore, no prominent fluorescence could be seen when pelleted cells were incubated with adipo-8, demonstrating that the aptamer probably did not bind to other cells within adipose tissue.

In conclusion, we have selected two aptamers, adipo-1 and adipo-8 that can recognize differentiated lipid-rich 3T3-L1 adipocytes by cell-SELEX with counter selection against preadipocytes and HepG2 cells. In particular, adipo-8 showed high specificity and affinity towards mature adipocytes with a K_d_ of 17.8±5.1 nM. Furthermore, adipo-8 showed no binding to any other cell line tested, indicating that this probe could be used to further characterize membrane protein expression after adipocyte differentiation. Further tissue slides test revealed that the adipo-8 can recognize adipose tissue, while no staining can be observed on tissue slides from rat liver, skeletal muscle, pancreas. These results indicate that this aptamer has the high possibility of binding to a molecular target specifically expressed in adipose tissue, especially adipocytes. Several strategies have been proposed for the treatment of metabolic disease via specific targeted delivery of therapeutic drugs to adipose tissue. Supported by our findings, this probe may also be exploited for direct targeted delivery of anti-obesity drugs to adipose tissue. Another interesting finding in this study is that little or no background fluorescence was observed when aptamers were incubated with frozen slides from untargeted tissue. Thus, performing SELEX on frozen tissue slides could be explored as a new strategy for the discovery of new tissue specific biomarkers.

## Materials and Methods

### Materials and Reagents

The 3T3-L1(Mouse 3T3-L1 preadipocytes), HepG2(liver hepatocellular cells), A549 (human lung adenocarcinoma epithelial cell line), Hct 116 cells(colorectal carcinoma HCT-116 cells), CK562 (human erythroid leukemia), Ramos (human Burkitt’s lymphoma cell line), PL45 (pancreatic cancer cells), MCF-7(breast cancer cells) and A172 (human glioblastoma cell line) were obtained from ATCC (American Type Culture Collection, Manassas, VA, USA). Human B cells and T cells were gifts from Lung-Ji Chang (Dept. of Molecular Genetics and Microbiology, University of Florida). C2C12 cells were kindly provided by Sally E. Johnson (Dept. of Animal Sciences, University of Florida). Dulbecco’s modified Eagle’s medium (DMEM), calf serum (CS), FBS, Oil Red O, phosphate-buffered saline (PBS), paraformaldehyde, bovine serum albumin (BSA), 3-isobutyl-1-methyxanthine (IBMX), insulin and dexamethasone (DEX) were purchased from Sigma (St. Louis, MO). Library and aptamers were synthesized on a 3400 DNA/RNA synthesizer (Applied Biosystems, Foster City, CA). The sequences were then deprotected in AMA (ammonium hydroxide/40% aqueous methylamine 1∶1) and purified by reversed-phase HPLC (ProStar, Varian, Walnut Creek, CA, USA). Taq-polymerase, dNTPs and other reagents were purchased from Takara. PCR was performed on a Biorad Thermocycler. Trypsin and Proteinase K were purchased from Fisher Biotech.

### Cell Culture and Staining

The 3T3-L1 preadipocytes were cultured in DMEM with 10% calf serum at 37°C in a 10% CO_2_ incubator. The cells were differentiated as described previously [38], [39]. Briefly, 3T3-L1 cells were grown to confluency in 10% calf serum/DMEM. Two days post-confluency (DAY 0), cell differentiation was initiated by adding MDI media (DMEM containing 10% FBS, 0.5 mM 3-isobutyl-1-methylxanthine, 1 µM dexamethasone, and 1.67 µM insulin). After 48 h (DAY 2), the incubation medium was replaced with insulin media (DMEM containing 10% FBS, 1.67 µM insulin). Two days later (DAY 4), the medium was changed to 10% FBS/DMEM. Cells were incubated with 10% FBS/DMEM every two days thereafter. Full differentiation was usually achieved by DAY 8. On DAYS 0, 4, 6, 8 and 10, cells were fixed with 4% formalin in PBS (pH 7.4) and stained with Oil Red O to evaluate the differentiation rate. Differentiated 3T3-L1 cells at DAY 10 were used for subsequent experiments. All other cell lines used for selectivity assays were cultured according to ATCC specifications.

### Cell-SELEX Procedure

The library of synthetic DNAs, composed of a central randomized sequence of 45 nucleotides flanked by two 20-nt primer hybridization sites (5′-ATGAGAGCGTCGGTGTGGTA-N45-TACTTCCGCACCCT CCTACA-3′), was amplified using a FITC-labeled 5′-primer (5′-FITC-ATGAGAGCGTCGGTGTGGTA-3′) and a biotin-labeled 3′-primer (5′-biotin-TACTTCCGCACCCTCCTACA-3′). Cell-SELEX was performed as previously described [40]. Briefly, the ssDNA pool (100 pmol) in 400 µL binding buffer was denatured by heating to 95°C for 5 min and then cooling on ice for 5 min for better folding. The ssDNA pool was then incubated with differentiated 3T3-L1 adipocytes at 4°C for 1 hour. After washing, cells were collected in 300 µL binding buffer in a 1.5 mL tube, and the bound ssDNAs were eluted by heating at 95°C. After centrifugation, the supernatant was incubated with undifferentiated 3T3-L1 preadipocytes or HepG2 cells for counter selection. After centrifugation, the supernatant was PCR-amplified with FITC- and biotin-labeled primers. The selected sense ssDNA was separated from the biotinylated antisense ssDNA strand by streptavidin-coated Sepharose beads (Amersham Pharmacia Biosciences) in 0.2 M NaOH. The purified ssDNAs were then used for the next screening library or for pool enrichment monitored by flow cytometry.

### Flow Cytometric Analysis

Mature adipocytes and undifferentiated precursors were first washed with PBS and detached with nonenzymatic dissociation buffer for 15 min. After washing, 50×10^4^ cells were incubated with FITC-labeled ssDNA pool dissolved in 200 µL binding buffer at a final concentration of 250 nM for 45 min. Cells were washed twice with 1 mL washing buffer and resuspended in 200 µL binding buffer. Fluorescence was determined with a FACScan cytometer (BD Immunocytometry Systems) by counting 30,000 events. The FITC-labeled unselected ssDNA library was used as a negative control. When the level of enrichment reached a plateau and no further shift could be observed, pools of interest were submitted for sequencing.

### Affinity Tests of Aptamers

The binding affinity of aptamers was determined by incubating differentiated 3T3-L1 cells (5×10^5^) on ice for 30 min in the dark with varying concentrations of biotin-labeled aptamer in a 200 µL volume. Cells were washed three times with 1000 µL WB, followed by resuspension in 150 µL BB containing streptavidin-PE for another 15 min. After washing, cells resuspended in 200 µL washing buffer were then subjected to flow cytometry, with 5′-biotin-labeled library as negative control. All of the experiments for binding affinity were repeated at least three times. The relative fluorescence intensity represents a ratio obtained through the following formula: relative fluorescensce intensity =  [(fluorescensce of aptamer - fluorescensce of library)/fluorescensce of library]. By using SigmaPlot (Jandel, San Rafael, CA), the equilibrium dissociation constants (K_d_) of the selected aptamers to cells were calculated by fitting the dependence of fluorescence intensity of specific binding on the concentration of the aptamers to the equation Y =  Bmax X/(K_d_ + X).

### Confocal Imaging of Cells Bound with Aptamer

For confocal imaging, the biotin-labeled aptamers and unselected control ssDNA pools were incubated with cells and washed as described for flow cytometry. After incubation with BB containing streptavidin-PE, cells were washed and subjected to confocal imaging with an Olympus FV500-IX81 confocal microscope. A 5-mW, 488-nm He-Ne laser was the excitation source for the phycoerythrins(PE) throughout the experiments. Twenty-five microliters of cell suspension bound with biotin-streptavidin-PE-labeled ssDNA were dropped on a thin glass slide placed above a 60x oil-immersion objective (PLAPO60XO3PH**)** with a numerical aperture of 1.40 (Olympus).

### Selectivity and Specificity

To determine the cell specificity of the selected aptamers, cell lines, including A549, Hct 116 cells, human B cells, T cells, CK562, Ramos, PL45, MCF-7, C2C12, and HepG2 cells, were used in binding assays by flow cytometry, as described above.

### Effect of Protease Digestion and Temperature on Aptamer Binding

Target cells (differentiated adipocytes) were detached using a nonenzymatic cell dissociation solution. After resuspension in WB, the cells were washed with 3 mL of PBS and then incubated with 1 mL of 0.05% trypsin/0.53 mM EDTA in HBSS or 0.1 mg/mL proteinase K in PBS at 37°C for 15 and 30 min, respectively. Pure FBS was added to quench the proteinases. After washing with 2 mL of BB, the treated cells were used for binding assays, as described above. The binding stability of selected aptamers was tested by incubating aptamers with adipocytes at 37°C, while aptamers incubated on ice were used as the positive control, followed by flow cytometry.

### Effect of Insulin on Binding Ability of Adipo-8

After serum starvation for 20 hours, mature adipocytes were incubated with DMEM media with 160 nM insulin for 10 min. Afterwards, cells were chilled on ice and incubated with biotin-coupled aptamer for 30 min. After washing, cells were incubated with streptavidin-PE dye for 15 min. After washing, cells were detached with nonenzymatic dissociation buffer for 20 min and used for flow cytometry.

### Staining of Rat Adipose Tissue Frozen Sections Using Selected Aptamers

Male Sprague-Dawley rats (n = 4, 200–250 g) were supplied by the Experimental Animal Center of Central South University. All procedures performed on the animals were in compliance with the Chinese Council of Animal Care guidelines. All experimental animals were approved by the Animal Care and Use Committee of XiangYa Hospital, Central South University. Rats were sacrificed by vertebral dislocation. After fresh segments of epididymal adipose, liver, skeletal muscle, or pancreatic tissue were frozen, they were embedded in optimal cutting temperature (OCT) compound, and transverse sections (10 µm) were generated with a cryostat, followed by mounting on charged glass slides. Sections were fixed with cold acetone for 10 minutes at room temperature and washed either for H&E staining or for fluorescence aptamer staining. For fluorescence aptamer staining, tissue sections were blocked with binding buffer containing 10% BSA and 1 mM tRNA at 4°C for 60 min and then dropped with 300 µL of 250 nM cy5-labeled aptamers in binding buffer. After incubation for 45 minutes, these tissue sections were washed three times with binding buffer. Finally, the stained sections were imaged by fluorescence microscopy (Nikon, Japan). The excitation wavelength was 633 nm, emission fluorescence was detected using a 670 nm filter, and the exposure time was 150 ms.

#### Preparation and incubation of adipose cells with selected aptamers for fluorescence microscopy

Male Sprague-Dawley rats were used to obtain primary adipose cells as described previously [37]. Briefly, the epididymal fat pads were removed and transfered to 5.5 mL prewarmed KRH buffer (120 mM NaCl, 5.2 mM KCl, 2.5 mM CaCl_2_, 1.2 mM KH_2_PO_4_, 1.2 mM MgSO_4_, 15 mM NaHCO_3_, 10 mM HEPES, 5.0 mM D-glucose, 3% BSA, pH 7.4). The fat pads were then minced with a sharp scissors for 2 min, and 15 mg collagenase type dissolved in 0.5 mL KRH buffer was then added to the mixture. After incubating in a shaking water bath with rapid shaking (120 cycles/min) for 60 min at 37°C, the cells were gently passed through stainless mesh (pore size 300 mesh). After centrifugation at 400 g at room temperature for 1 min, adipocyte (floating cells) and pelleted cells were resuspended separately in 30 mL fresh buffer (37°C). The centrifugation and washing were repeated three times. The cells were resuspended in 3 mL binding buffer (approx 2.5×10^6^ cells/mL). For fluorescence microscopy, the cy5-labeled aptamers and unselected control ssDNA pools were incubated with isolated rat adipose cells. The infranatant was withdrawn, and the floating cells were washed three times and used for fluorescence microscopy. The pelleted cells were incubated and washed as described for flow cytometry. Twenty-five microliters of cell suspension incubated with cy5-labeled aptamers or ssDNA were dropped on a thin glass slide and subjected to fluorescence microscopy as described above with an exposure time of 150 ms.

## Supporting Information

Figure S1 Dot plots of side scatter versus forward scatter of 3t3-L1 cells generated from flow cytometric analysis of levels of granularity at 0, 10 days after induction. The region above the bar was gated to include the differentiated 3T3-L1 cells.
(TIF)

Figure S2 Flow cytometry assay of selected pool with negative HepG2 cells.
(TIF)

Figure S3 The binding curve of adipocytes incubated with varying concentrations of PE-Cy5-labeled adipo8 aptamer(A), PE-Cy5-labeled adipo1(B) and their unselected library. (Error bars: SD, N = 3).
(TIF)

Figure S4 (*Upper*) H&E staining of frozen slides of liver (A), skeletal muscle, (B) and pancreatic tissue (C) from SD rat. (*Lower*) 250 nM adipo-8 applied to frozen slides of different tissues. (*Lower*. Images were obtained at 100× magnification.
(TIF)
