# Supplementary figures and images for: Selection of Aptamers Specific for Adipose Tissue
Source: PLoS One. 2012 May 25;7(5):e37789. doi: 10.1371/journal.pone.0037789 (PMC3360593; doi:10.1371/journal.pone.0037789)

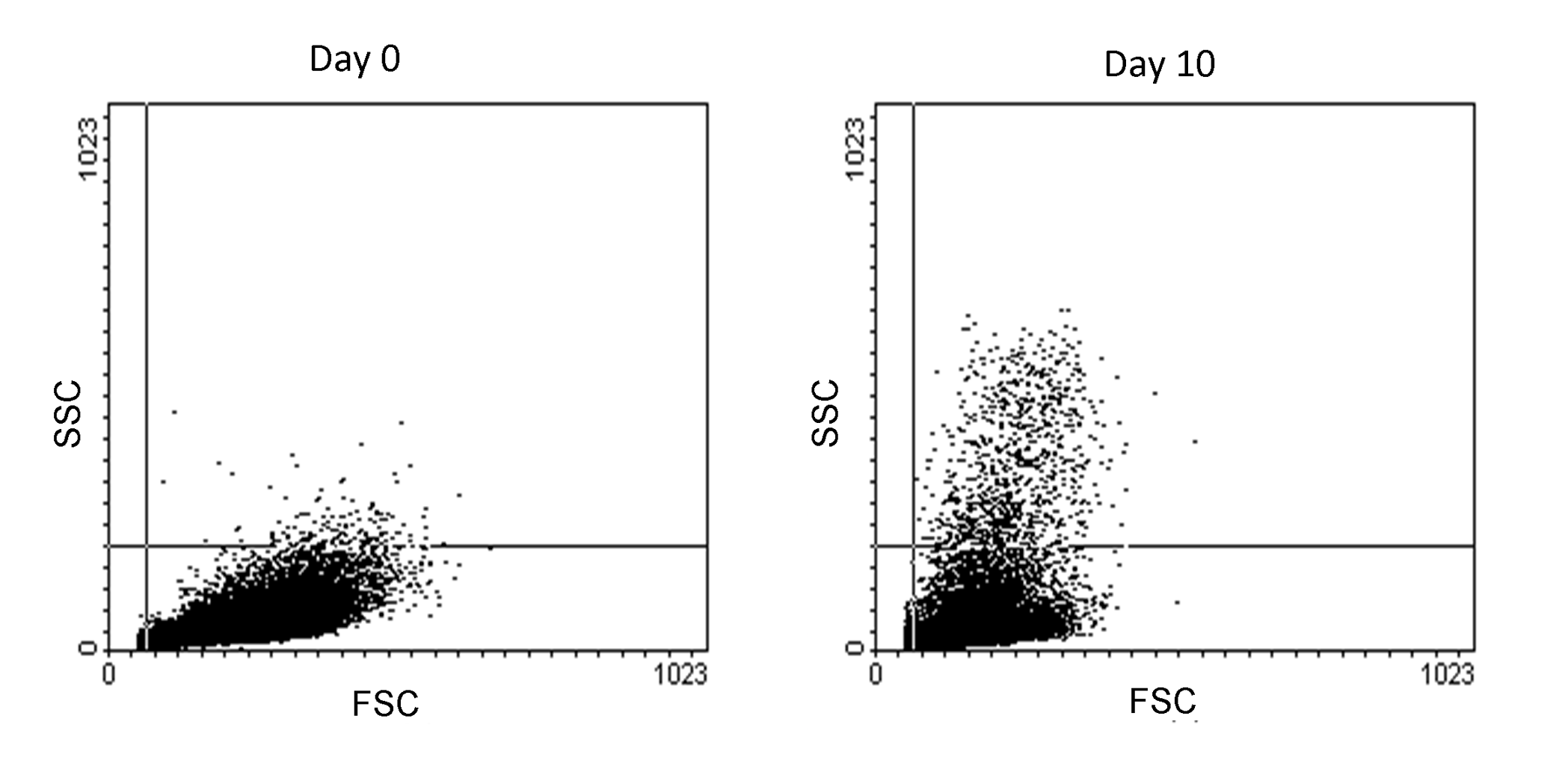

Supplement: Figure S1 — Dot plots of side scatter versus forward scatter of 3t3-L1 cells generated from flow cytometric analysis of levels of granularity at 0, 10 days after induction. The region above the bar was gated to include the differentiated 3T3-L1 cells. (TIF) [file pone.0037789.s001.tif]

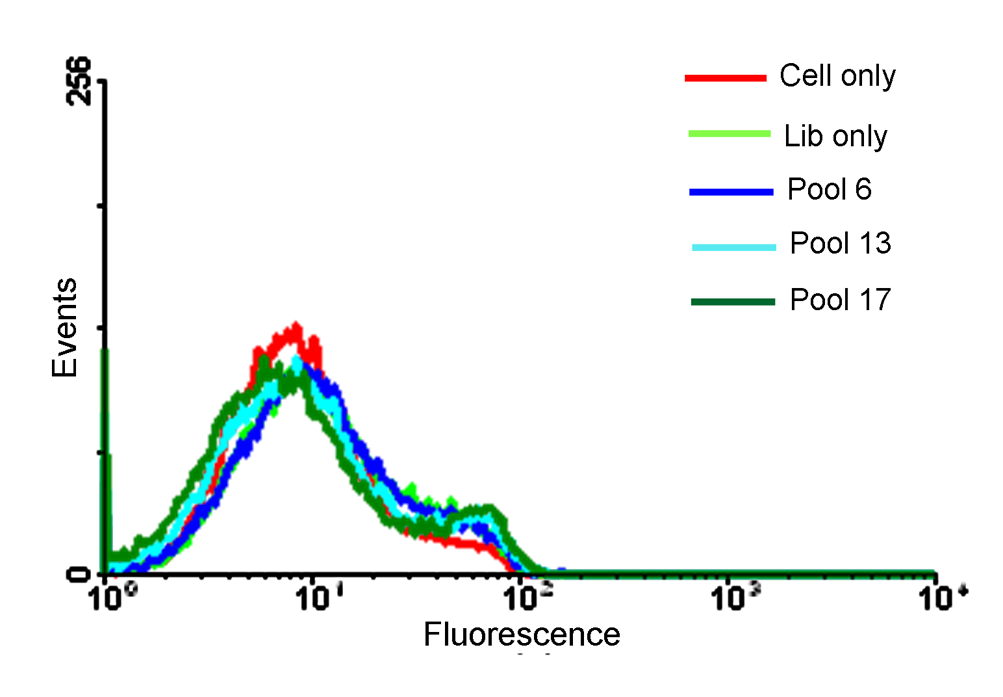

Supplement: Figure S2 — Flow cytometry assay of selected pool with negative HepG2 cells. (TIF) [file pone.0037789.s002.tif]

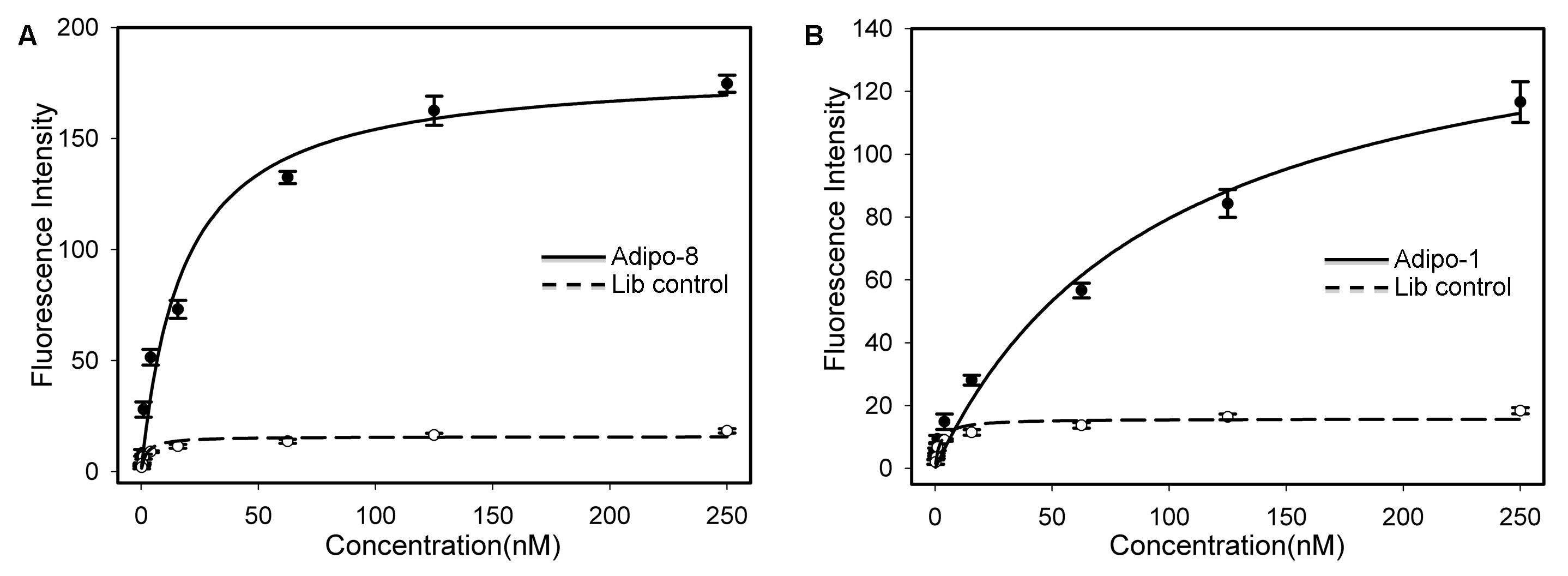

Supplement: Figure S3 — The binding curve of adipocytes incubated with varying concentrations of PE-Cy5-labeled adipo8 aptamer(A), PE-Cy5-labeled adipo1(B) and their unselected library. (Error bars: SD, N = 3). (TIF) [file pone.0037789.s003.tif]

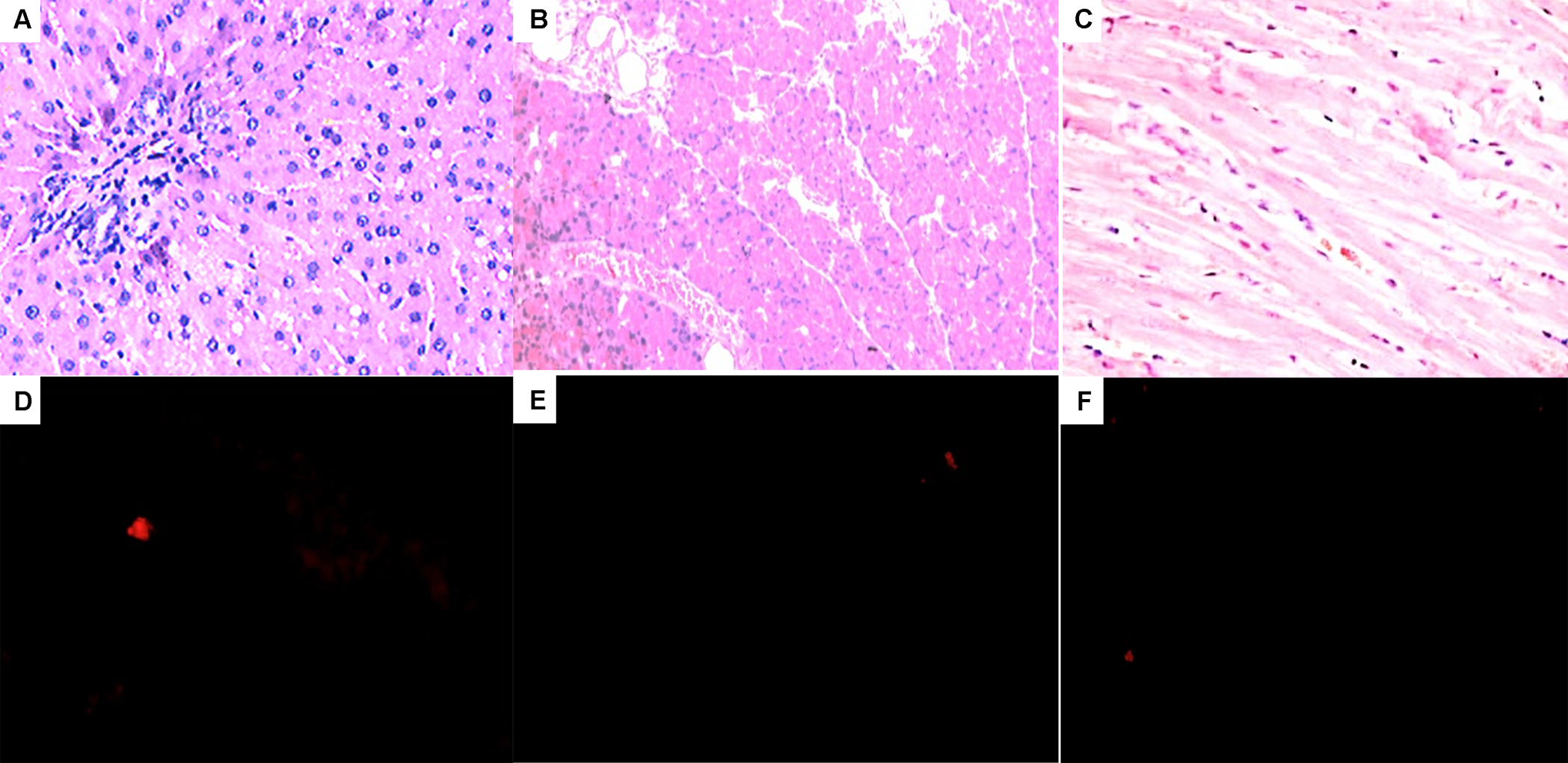

Supplement: Figure S4 — (Upper) H&E staining of frozen slides of liver (A), skeletal muscle, (B) and pancreatic tissue (C) from SD rat. (Lower) 250 nM adipo-8 applied to frozen slides of different tissues. (Lower. Images were obtained at 100× magnification. (TIF) [file pone.0037789.s004.tif]
